# Progress in Surgery

**Published:** 1896-08-22

**Authors:** 


### Progress in Surgery.

### SURGERY OF THE VERMIFORM APPENDIX.

**Appendicitis**, says Dieulafoy,[1] is invariably the result of the conversion of the appendicular canal into a closed cavity. This may take place at any point of the canal, and is accomplished in various ways. As a rule, there is partial obliteration, due to slow and gradual formation of a calculus, which results from a genuine appendicular lithiasis, comparable to biliary and renal lithiasis, and which the author regards as belonging to the patrimony of gout and arthritis. At other times, the conversion of the appendicular canal into a closed cavity is the result of a local infection, comparable to the changes occurring in the bile ducts in so-called catarrhal jaundice. Lastly, in some cases, this conversion results from the slow and gradual formation of fibrous stenosis, similar to constriction of the urethra. Several of these causes may co-exist. Symptoms of appendicitis do not supervene until the conversion of the appendix into a closed cavity is completed. The microbes existing in the appendix, which before have been harmless, then multiply and become virulent. The virulence may be such that the patient may die from this cause alone, with scarcely any perceptible lesion. At other times, the infection is propagated from the appendix to the peritoneum without a sign of perforation of the walls of the appendix. Lastly, the appendicular infection may end in gangrene and perforation of the appendix, followed by the evolution of the different varieties of perforative peritonitis. Le Dentu[2] thinks that when there is a distinct inflammatory reaction, definitely localised in the cœcal region, and when the course of the disease remains normal, without any indication of serious danger threatening, it is advisable to let the abscess definitely form before intervention. In such cases a very early operation may result in dispersing the infective agents, hitherto concentrated at a limited spot. On the contrary, the moment it has become evident that the purulent focus has formed, operation becomes opportune and imperative. Among cases terminating in circumscribed suppuration there are some which the anterior situation of the abscess renders benign, and others in which its posterior or deep situation is a source of danger. In the latter there is no occasion for reproving oneself for not having intervened at the onset, inasmuch as there was nothing threatening in the initial symptoms. Operation is urgently demanded: (1) When the disease at once takes the form of generalised peritonitis, or when, in the course of appendicitis, signs of extension to the entire peritoneum supervene. These cases must not be confused with those in which symptoms of "peritonism" exist. (2) When depression is manifested by such symptoms as absence of spontaneous pain, normal or nearly normal temperature, with small and extremely rapid pulse, defective urinary secretion, retracted abdomen, and cyanotic condition of the extremities. (3) It is dangerous to abstain from operation when the morbid symptoms recur. Unless the attacks come too close together, it is well to wait five or six weeks, when most of the exudation will be found to have disappeared, and the operation becomes a very simple one. Meyer[3] considers that the indications for operation are as follow: (1) In cases of diffuse perforative appendicitis the operation must always be done at once. Patients have the best chance to recover, if operated upon within the first twelve hours, although exceptionally they get wel without operation. (2) In cases of acute appendicitis the patient always needs careful observation. If the pulse goes up above 116 to 120, and has a tendency to stay there, the indication for an operation is given. The temperature is not of value in deciding the question of operation. In case of doubt, the operation is better than waiting. (3) In cases of subacute (mild) attack of appendicitis, also after the first severe attack, from which the patient recovers without immediate operation, the appendix should be removed. The appendix, once inflamed, has to be looked upon as a diseased organ which is very apt to give repeated and more serious, even fatal, trouble in the future. Kime[4] thinks that by the use of salines and disinfectants many patients can be relieved by unloading the colon, and diminishing the hyperæmia. If a patient progresses satisfactorily for some days and then becomes seriously worse, an operation should be done without delay. The appendix should not be removed when an abscess cavity is opened which is walled off from the general peritoneal cavity. Royster,[5] quoting Price, says that recovery without operation is probable—(1) If nausea disappears in twelve hours; (2) if tenderness on pressure is not increased in twelve hours; (3) if the temperature is normal or not above 100 deg. Fahr. in the mouth; (4) if the pulse is normal or but little accelerated; (5) if the patient moves in bed with ease. Coley[6] narrates an interesting case of appendicitis due to traumatism in a boy of sixteen who had a distinct tumour in the right iliac region, due to a blow with the handle of a push cart three days before. The temperature rose to 104 deg. Fahr., and on operation an abscess, about the size of a walnut, was found just within the abdominal wall, and the tip of the appendix was gangrenous. Robinson[7] thinks that before the operation stage arrives, many cases can be cured by anti-rheumatic treatment (salicin, salol), and refers to the large amount of lymphoid tissue in the appendix, pointing to similarity of structure with the tonsils which are known to reflect the rheumatic tendency. Mild laxatives, not active purgatives, are beneficial. Opium is harmful, not only because it obscures the symptoms, but also because it dries up the secretions. Deaver[8] once more emphasises his opinion that early operation is the safest road to recovery, and in support of this view he states that he has now operated upon two hundred cases with only two deaths, and those in the first hundred. He maintains that there are three unmistakable cardinal symptoms:—1. The sudden onset of acute abdominal pain, with or without vomiting, occurring in one who was previously well. 2. Rigidity of the right side of the lower abdominal wall. 3. Tenderness over the site of the appendix. Bellamy[9] narrates a case which was operated upon by Kocher in two stages. Finding the abscess was not shut off from the general peritoneal cavity by omental and mesenteric adhesions, no attempt was made to open the abscess or remove the appendix at the first operation, but the wound was plugged with iodoform gauze. Seven days later, when an artificial wall had formed, the abscess was opened and the appendix removed. Morris[10] believes that the neurasthenia which sometimes appears after an operation for appendicitis, while not peculiar to these cases, is quite characteristic of them. He believes it to be due to toxæmia. Bellamy[11] has had three cases of neurasthenia following operations on the appendix under his care. Roux[12] has operated upon 95 cases of recurrent appendicitis, between the attacks, removing the appendix about five or six weeks after the last attack. He thinks that in spontaneous cure about 50 per cent. of cases recover by evacuation of the pus into the small intestine (hence its absence from the stools), and the rest by absorption of the pus which he has observed in every grade of thickening, caseation, and calcification. He found adhesions in every case. One patient died from embolism after perfect operative recovery.

[1] The Med. Week, Feb. 14th, 1896. [2] Ibidem, March 27th, 1896. [3] Med. Rec., New York, Feb. 29th, 1896. [4] New York Med. Journ., Jan. 28th, 1896. [5] Charlotte Med. Journ., Feb., 1896. [6] Med. Rec., New York, Feb. 15th, 1896. [7] Ibidem. [8] Practitioner, March, 1896, and Med. News, Dec. 21st, 1895. [9] New York Med. Journ., March 21st, 1896. [10] Ibidem. [11] Ibidem. [12] Amer. Med.-Surg. Bulletin, Jan. 11th, 1896.

